# The Possible Remedial Measures

**Published:** 1920-03-13

**Authors:** 


### ANATOMY ACT REFORM.

### II. The Possible Remedial Measures.

### The Present Crisis.

At the commencement of the present academic term the shortage of bodies was so acute at one London Medical School that for over sixty second- and third-year students ready to dissect, only one subject was available; in consequence a series of demonstrations were held, the students witnessing a skilled dissection instead of performing this most useful duty for themselves. The task of dissection is as much an art as a science, and much of the digital delicacy and dexterity of the embryo surgeon is attained in this way. At another London hospital subjects for dissection were so scarce that the students worked in relays, and so shared the limited material between them.

The only teaching of operative surgery of any great value is the experience of students actually performing operations, under their teachers' directions, on the cadaver itself. So important is this surgical training, that the conjoint examining board of England prescribe that at least twenty operations be performed in this manner before a candidate can have his schedule signed to be allowed to sit for his final examination, whilst the examination for the final Fellowship of the Royal College of Surgeons of England is formed partly of an actual examination of the candidates in the art of operative surgery on the dead body.

At the present time no bodies are available for the practice of operative surgery, as all material possible has to be conserved for the still more important teaching of anatomy. Surgery rests upon anatomy as a foundation, and cannot exist as a separate entity.

### Alterations forced on the Examining Boards.

Dental students have been precluded from any actual dissections whatever, and the examining boards have had temporarily to alter their syllabus in consequence.

At the forthcoming examination in May of the Royal College of Surgeons for the final fellowship, the customary examination in practical surgery, which in the past has been one of those factors which has made this qualification *facile princeps* in the surgical world, has perforce to be omitted.

Now all these difficulties are hindering the onward progress of surgery, and unless they be removed the profession and the general public must reconcile themselves to a lower average standard of surgical efficiency in the newly-qualified medical man.

### The Requirements for Anatomy and Surgery.

The minimum requirements of the anatomical authorities at the present time are one subject per student entering the medical or dental professions each year.

The present sources of material, as prescribed by the existing Anatomy Act, are the unclaimed bodies of those dying in pauper institutions. An interview with one of the Professors of Anatomy of London University revealed the fact that ample material is actually in existence in such sources at the present time, but it appears that the Anatomy Act, in common with the Public Health Act and the ill-fated Dental Act, lacks its full effect through a failure to carry into being the full meaning and spirit of the measures it provides.

### The Root of the Trouble.

Under the existing Anatomy Act, when a person dies in a pauper institution and the body is unclaimed within twenty-hour hours, it is at the disposal of the Board of Guardians to bury it at the public expense, or else to transfer the body to the nearest school of anatomy, at the expense of the school.

Unfortunately, a meeting of the Guardians is requisite for the latter course to be taken, and so in order to save themselves the trouble of such a meeting, or in obedience to the views of certain of their number against dissection, the body in the majority of instances is buried at the public expense, against the meaning of the Anatomy Act, and against both the interests of the medical profession and the general public.

### The Remedy.

The Anatomical Society of Great Britain and Ireland have interviewed Dr. Addison, the Minister of Health—himself for many years a professional anatomist—and the present policy of the Ministry towards this question will be to circularise and interview the Boards of Guardians who hinder the smooth working of the Anatomy Act. If the Guardians yield to the advice of the Ministry, then all will be well; for if *all*, and not a proportion, of the unclaimed bodies are conveyed to the schools of anatomy, then present needs will be amply supplied.

On the contrary, if the Guardians prove obdurate, then nothing remains but to seek new legislation, a measure being sought which will allow the anatomical schools to remove *all* unclaimed bodies.

This is the only way, the only possible way, by which the present generation of medical men can attain, for the service of the public, that surgical skill and knowledge which was the heritage of their forbears for nearly a century.

We have every confidence that ultimately a satisfactory arrangement will be made, as our Government has shown itself sufficiently alive in health matters not completely to miss the opportunity which the actual existence of material provides.

The previous article appeared on February 21, p. 484.

